# Therapeutic Antitoxoplasmosis Potential of *Garcinia cambogia*: Evidence From In Silico and In Vivo Studies

**DOI:** 10.1155/jotm/7285376

**Published:** 2026-09-21

**Authors:** Marwa M. I. Ghallab, Eman S. El-Wakil, Rabab S. Zalat, Mina A. Almayouf, Hayat S. Al-Rashidi, Wafa Abdullah I. Al-Megrin, Salwa M. Morsy

**Affiliations:** ^1^ Department of Medical Parasitology, Faculty of Medicine, Kafrelsheikh University, Kafrelsheikh, Egypt, kfs.edu.eg; ^2^ Department of Parasitology, Theodor Bilharz Research Institute, Giza, Egypt, tbri.sci.eg; ^3^ Department of Biology, College of Science, Qassim University, Buraydah, Saudi Arabia, qu.edu.sa; ^4^ Department of Biology, College of Science, Princess Nourah bint Abdulrahman University, Riyadh, Saudi Arabia, pnu.edu.sa; ^5^ Department of Medical Parasitology, Faculty of Medicine, Cairo University, Giza, Egypt, cu.edu.eg; ^6^ Department of Medical Parasitology, Faculty of Medicine, Modern University for Technology and Information, Cairo, Egypt, mti.edu.eg

**Keywords:** *Garcinia cambogia*, guttiferone, histopathology, hydroxycitric acid, Iba1, parasitism rate, *Toxoplasma gondii*

## Abstract

*Toxoplasma gondii*, an opportunistic protozoan, is widely prevalent and poses potential health risks. Its treatment remains challenging with several side effects, urging the exploration of new, safe, and efficient drugs. We aimed to explore the therapeutic efficacy of the medicinal plant *Garcinia cambogia* (GC) against both acute and chronic toxoplasmosis. Sixty Swiss albino mice were allocated into five groups: I (healthy), II (model), III (spiramycin [SPM] treated, IV (GC treated), and V (SPM + GC treated). Each group is further divided into two subgroups receiving treatment at 4 and 42 days postinfection (dpi) for acute and chronic infection, respectively. To determine the properties of the GC active compounds, hydroxycitric acid (HCA), guttiferone‐E, guttiferone‐J, compared to SPM against the selective *T*. *gondii* ROP18 transferase target regarding its binding affinities, and estimated inhibition constant, Lipinski’s RO5 filter analyses and computational QSARs/ADMET predictive characteristics were used. The groups were evaluated parasitologically, histopathologically, and immunohistochemically for Iba‐1 expression. The best treatment outcome was observed in the SPM + GC–treated groups in both acute and chronic infections, with inhibition percentages of 89% and 78%, respectively, and preservation of neuronal integrity, as evidenced by the lowest Iba1 expression levels. Moreover, the docked ROP18‐HCA revealed an additional, critical, and novel ligand‐binding site for ROP18, which is essential for the virulence and pathogenesis of *Toxoplasma*. *G. cambogia*’s stability of intermolecular binding activities, potency, and conformations makes it an effective, safe, and promising remedy for treating toxoplasmosis, especially in combination with SPM.

## 1. Introduction

The obligate intracellular apicomplexan protozoan *Toxoplasma gondii* infects a wide range of animals, including those with worm‐like bodies, as well as humans, with prevalence varying significantly across countries. It is estimated that more than one billion people may be infected with *T. gondii* [1].

Infection may be subclinical, manifest as ocular affection, or present as cervical lymphadenopathy. However, severe cases may present multiple health consequences that may be life‐threatening, with dormant infection linked to the pathogenesis of many neuropsychiatric disorders [2].

Toxoplasmosis treatment involves a combination of dihydrofolate reductase inhibitors and sulfa drugs. Still, these regimens can cause adverse side effects that hinder their use, such as rash, hypersensitivity, hematologic toxicity, and bone marrow suppression. Moreover, this regimen is lethal to active tachyzoites but does not affect dormant bradyzoites [3]. Given the critical and challenging therapeutic regimen, which in turn affects disease outcomes, the discovery of new therapeutic alternatives with low toxicity and fewer side effects is prioritized [1].

Natural products are considered promising alternatives for drug development. *Garcinia cambogia* (GC) is a small‐ to medium‐sized Southeast Asian tree. Both the rind and the pulp contain a chemical called hydroxycitric acid (HCA), a dimeric flavonoid, benozophenones, organic acids, and over 68 xanthones, particularly α‐, β‐, and γ‐mangostin. Numerous studies have demonstrated that *Garcinia* species possess anti‐inflammatory and antioxidant properties, stimulate both cellular and humoral immunity, and are effective antiparasitic agents [4]. In this context, *G. celebica* leaves have been shown to inhibit the growth of *Plasmodium falciparum* [5]. The ethanol extract of *G. mangostana* Linn was tested against *Acanthamoeba*, showing a marked decrease in parasitic load [6]. The potential leishmanicidal effect of *G. cowa* against Leishmania donovani has been confirmed in prior research, and it has been recommended as a potential drug candidate for clinical trials [7].

Currently, promising and selective multitarget antiparasitic phytocompounds are being identified to evaluate their therapeutic efficacy and potential drug targets, thereby reducing the harmful effects of conventional treatment protocols [8]. For drug discovery, design, and development, the identification of promising chemical ligand targets is a significant advance. Molecular docking simulations are widely used to identify ligands, explore new treatment avenues by visualizing and predicting binding modes and poses, and assess binding affinities for specific protein targets [9].

Based on this, we attempted to evaluate the impact of GC in the treatment of acute and chronic murine toxoplasmosis. Additionally, the drug‐likeness properties of HCA, guttiferone‐E, and guttiferone‐J were predicted, determined, and evaluated using analyses of Lipinski’s rule of five (RO5) filter, computational QSARs/absorption, distribution, metabolism, excretion, and toxicity (ADMET) predictive characteristics, and in silico molecular docking compared to spiramycin (SPM) as a conventional synthetic standard antiparasitic drug. Besides, the potent restorative therapeutic features of these compounds as antitoxoplasmosis ligands against the toxoplasmositic properties of *T*. *gondii* ME49 were detected using the structure‐based (SB) and in silico ligand‐based (LB) virtual screening analyses via visualizing and assessing their affinities, binding models, and noncovalent intermolecular interactions toward *T*. *gondii* ME49–selected target, such as rhoptry kinase family protein (ROP18 transferase), compared to SPM.

## 2. Materials and Methods

### 2.1. The Selection of Computational Tools

In the early stages of drug discovery, computational tools are often used to rank potential compounds before thorough experimental validation [10]. In the current study, the computational workflow included multiple tools chosen for their demonstrated predictive performance, algorithmic robustness, and benchmarked applicability. Molecular docking was used because SB docking to the ROP18 kinase, a well‐defined parasitic protein, is a standard method for exploring binding modes and affinities and for prioritizing small‐molecule candidates, in line with published virtual screening and inhibitor discovery studies targeting ROP18 [11]. Lipinski’s RO5 and drug‐likeness filters were applied to screen for physicochemical properties compatible with oral drug development and to serve as empirical predictors of absorption and permeability [12]. QSAR and ADMET predictions were incorporated to estimate pharmacokinetic and toxicity profiles and to complement docking results, with integrated docking–QSAR–ADMET pipelines helping to prioritize promising hits before labor‐intensive in vitro and in vivo testing [13].

### 2.2. QSARs/ADMET In Silico Predictive Characteristics of HCA, Guttiferone‐E, Guttiferone‐J, and SPM

First, the PubChem server was used to retrieve the canonical SMILES of HCA, guttiferone‐E, guttiferone‐J, and SPM and accessed at https://pubchem.ncbi.nlm.nih.gov/. Then, we estimated their characteristics (ADMET). In this research, we targeted HCA, guttiferone‐E, guttiferone‐J, and SPM for predicting their molecular and physicochemical characteristics using multiple ADMET web tools, including admetSAR2 (https://lmmd.ecust.edu.cn/admetsar2), ProTox‐II (https://tox-new.charite.de/), Molinspiration Cheminformatics software Version 2022.08 (https://www.molinspiration.com), and SwissADME (https://www.swissadme.ch/). Afterward, we evaluated the pharmacokinetic (ADMET) potentials, lipophilicity indices, physicochemical properties, medicinal chemistry friendliness, and biological drug‐likeness scores of HCA, guttiferone‐E, and guttiferone‐J compared with those of SPM.

### 2.3. SB In Silico Virtual Screening Analysis of HCA‐, Guttiferone‐E‐, Guttiferone‐J‐, and SPM‐Correlated *T*. *gondii* ME49 ROP18 Complexes

#### 2.3.1. Preparation and Optimization of the Selective GC Phytochemicals, SPM, and the ROP18 Target

The canonical SMILES of (−)‐HCA (or garcinia acid, MF: C_6_H_8_O_8_, MW: 208.12 g/mol, CID:185620), (−)‐guttiferone‐E (camboginol/carcinol/polyisoprenylated benzophenone, MF: C_38_H_50_O_6_, MW: 602.81 g/mol, CID: 6245985), guttiferone‐J (garciyunnanin A, MF: C_38_H_50_O_5_, MW: 586.81 g/mol, CID: 102031302), and SPM (foromacidin A/formacidine/rovamycin, MF: C_43_H_74_N_2_O_14_, MW: 843.05 g/mol, CID: 5289394) were gathered using the PubChem database (https://pubchem.ncbi.nlm.nih.gov/). For ligand preparation. Additionally, the ChemSketch application from ACD Laboratories, Version 12.0, was used to create, optimize, and clean the chemical structures of HCA, guttiferone‐E, guttiferone‐J, and SPM. These were stored in MDL MOL file format (.mol). Furthermore, these ligands were converted to PDB format (pdb) from MDL MOL using the Open Babel GUI v2.3.2 and then energy‐minimized.

The x‐ray crystallographic structure of *T. gondii* ME49 rhoptry kinase family protein (ROP18 transferase/ROP18 kinase domain, PDB ID: 4JRN, 2.71 Å) [14] was retrieved in PDB‐file format from the RCSB‐PDB database (https://www.rcsb.org/) for protein target preparation. For energy minimization, the selected anti‐*T*. *gondii* target protein has been processed using the Swiss‐PDBViewer Version 4.1.0 software. The PROCHECK/Pdbsum web server’s Ramachandran plot analysis was used to assess the quality of the modeled proteins (https://www.ebi.ac.uk/thornton-srv/software/PROCHECK/) [15].

#### 2.3.2. Molecular Docking Simulations

The binding affinities (kcal/mol) and estimated inhibition constants (Ki) of HCA, guttiferone‐E, guttiferone‐J, and SPM against their antitoxoplasmosis ROP18 target were determined using molecular docking with Autodock v4.2.6 software. The ligands and chosen root structures were detected for lead optimization and saved in PDBQT format. Heteroatoms, water molecules, and complex moieties were eliminated to optimize the ROP18 protein. Subsequently, in pdbqt formats, Gasteiger and Kollman charges and polar hydrogens were included.

#### 2.3.3. Grid Box Generation and Processing of AutoGrid4 and AutoDock4

Target proteins were used as the grid box centers with 0.375 Å spacing, grid box values *X* = 0.757, *Y* = 45.069, and *Z* = 23.971, and npts = 160 × 160 points across the X‐, Y‐, and Z‐dimensions to define the active catalytic‐binding sites. A Lamarckian genetic algorithm (GA) was used to determine the optimal docked conformation, and 10 GA runs were conducted with the following parameters: a population size of 150, 25,000 energy evaluations, and 27,000 generations. We used a root‐mean‐square deviation (RMSD) of 2.0 Å to cluster the 10 conformations. Then, the pdb‐file format was used to save the conformation/binding mode with the lowest energy. The selective target displayed a specific scoring function toward its lead if the binding affinity is less than −5 kcal/mol [16, 17]. The negative values of the estimated free energy for target–ligand‐docked complexes are positively correlated with their binding affinities and, subsequently, their molecular docking features. The binding affinities (Kcal/mol) showed the lowest negative values for the optimal binding modes [18].

### 2.4. Visualization of the HCA‐, Guttiferone‐E–, and SPM‐Correlated Selective Antitoxoplasmosis ROP18 Complexes and Demonstration of Their Noncovalent Intermolecular Interactions

The BIOVIA Drug Discovery Studio Visualizer program was used to view and assess the binding mode with the highest binding affinity for the ligand‐docked antitoxoplasmosis ROP18 complex.

### 2.5. Ethical Considerations

With approval code MKSU 50‐11‐24, the study’s ethical approval was authorized by the ethics committee of Kafrelsheikh University’s Faculty of Medicine, which was then performed according to the criteria of the Clinical and Laboratory Standards Institute (CLSI) and adhered to the ARRIVE guidelines.

### 2.6. Animals

The current research utilized 60 Swiss albino CD1 male mice from the Theodor Bilharz Research Institute (TBRI) in Giza, Egypt. The mice weighed 20–25 g on average and were 6–8 weeks old. Mice were kept in air‐conditioned rooms (21 ± 2°C) in hygienic, well‐ventilated plastic cages, away from direct sunlight, and with unrestricted availability to water and food.

Using G∗Power 3.1.9.7 [19], an a priori power analysis was carried out. Setting *α* = 0.05, power (1‐*β*) = 0.90, and a large effect size (*f* = 0.70) for a one‐way analysis of variance (ANOVA) with 10 groups, the analysis showed that a sample size of 50 was necessary. The sample size was adjusted to *N* = 60 to account for an estimated 20% dropout rate.

### 2.7. Infection

The cytogenic *T. gondii* Me49 strain was gathered from the animal house of TBRI. It was preserved by repeatedly injecting 100 μL of brain homogenate via an esophageal tube every 8 weeks into Swiss albino mice that had already contracted the infection, with approximately 1 × 10^2^ tissue cysts per mouse [20].

After homogenizing the mice’s brains in sterile saline containing 1% penicillin/streptomycin, the infective dose was adjusted to about 100 cysts/mouse. By oral gavage, mice from the infected and infected treated groups were infected at 0 days postinoculation (dpi) [2, 21].

### 2.8. Experimental Design

The mice were randomly assigned to one of two primary groups: acute toxoplasmosis treatment (Division A), in which mice received treatment at 4 days postinfection (dpi), and chronic toxoplasmosis treatment (Division B), in which mice received treatment at 42 dpi. Every division was further subdivided into the five groups listed below (I–V), each of which had six animals:

 Group I (healthy): noninfected nontreated group (healthy mice),
 Group II (model): infected nontreated group (model mice),
 Group III (SPM): infected and treated with SPM (Pharaonia Pharmaceuticals, Egypt) at a dose of 200 mg/kg body weight/day by oral gavage for 2 weeks [22–24],
 Group IV (GC): infected and treated with GC (Super Citrimax, Natrol Inc., Chatsworth, CA 91311, USA) at a dose of 200 mg/kg body weight/day by oral gavage for 2 weeks [25, 26].
 Group V (SPM + GC): infected and treated with both SPM and GC*.* The combined treatment group follows the same dosing and timing as when each drug is given alone.

Additionally, the study was blinded, with codes assigned to animal groups and medications used during treatment that were only disclosed after euthanasia.

### 2.9. Parasitological Evaluation

Mice were euthanized by light isoflurane through the inhalation route 24 h after the last treatment (18 dpi for Division A and 56 dpi for Division B) [27].

Each mouse’s brain was removed after it was sacrificed. Each brain half was rinsed in PBS and then homogenized in PBS (1 mL) for 5 min. Three 20‐μL aliquots of the brain suspension were analyzed on microscope slides. Cysts of *T. gondii* were counted and recognized morphologically under a microscope. The cyst concentration per milliliter of brain solution was computed as the average of the counts [28]. Then, the following formula was utilized to determine each drug’s inhibition percentage: inhibition (%) = C‐T/C × 100, where C is the mean cyst count of the infected untreated group, and T is the mean cyst count of the infected treated group [29].

### 2.10. Histopathological Evaluation

Each mouse had half of its brain tissue preserved in 10% formalin, then dehydrated, cleared, and embedded in paraffin blocks. Light microscopy was used to analyze 5‐μm‐thick brain sections after preparation and H&E staining [30].

### 2.11. Immunohistochemical Staining and Evaluation

Immunohistochemical staining was performed according to the manufacturer’s procedures. Sections of antigen‐retrieved brain tissue were treated with hydrogen peroxide (0.3%) for 15 min to suppress endogenous peroxidase. Following that, the anti‐Iba1 ionized calcium‐binding adapter protein 1) antibody (ab108539—Abcam, 1:100) was incubated overnight at 4°C. This was followed by a PBS wash and a 20‐min incubation with the HRP EnVision secondary antibody kit (DAKO). Before the 10‐min diaminobenzidine incubation, a PBS wash was performed. After dehydration, xylene cleaning, and hematoxylin counterstaining, tissue sections were cover‐slipped for microscopic inspection.

For quantification, three nonadjacent coronal sections from each animal’s brain were analyzed. Five to eight nonoverlapping fields from each section were randomly selected within the cerebral cortical regions and analyzed using the Leica Application System histological analysis modules (Leica Microsystems GmbH, Wetzlar, Germany). Fields were sampled using a systematic random approach, excluding areas with artifacts, and all images were acquired under standardized illumination and exposure settings by an observer blinded to group allocation. The images were processed to isolate the DAB signal, and a fixed optical intensity threshold, calibrated on representative sections and verified with negative controls without primary antibody, was applied uniformly across all groups. The relative mean positive area percentage was calculated as follows: (Iba1‐positive pixels area/total pixels area) × 100, and values were averaged per animal [31, 32].

### 2.12. Statistical Analysis

GraphPad Prism 10.4.0 (GraphPad Software, San Diego, CA, USA) was used for statistical analysis. First, data distribution was assessed using the Shapiro–Wilk test to determine normality. Accordingly, normally distributed data are presented as mean ± standard deviation (SD), whereas non‐normally distributed data are expressed as median with interquartile range (IQR). Multiple group comparisons were conducted using one‐way ANOVA, followed by Tukey’s post hoc test, for normally distributed data. For non‐normally distributed data, the corresponding nonparametric tests were applied as appropriate. Statistical significance was set at *p* < 0.05.

To investigate the relationship between parasitological burden and neuroinflammatory activation, correlation analyses were performed between tissue cyst count (*X* variable) and the percentage of Iba1‐positive area (*Y* variable) in both acute and chronic infection models. Spearman’s rank correlation coefficient was used, and the results are presented with corresponding 95% confidence intervals. Scatter plots were generated to visualize these associations.

## 3. Results

### 3.1. Predictive QSARs/ADMET In Silico Properties of Selective GC Active Components, Such as HCA, Guttiferone‐E, and Guttiferone‐J, Compared to SPM

**FIGURE 1:**
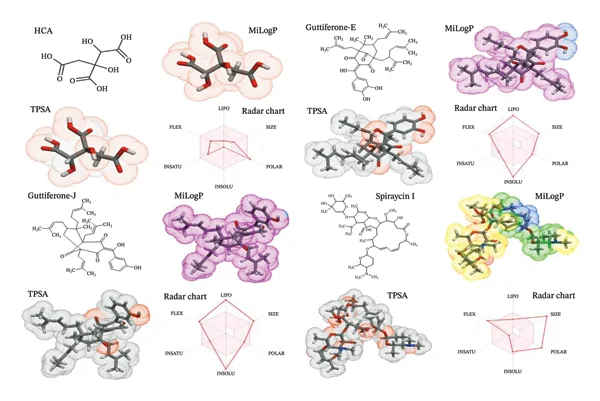
The 2D‐cleaned chemical structures of HCA, guttiferone‐E, guttiferone‐J, and spiramycin were optimized using ACD Labs ChemSketch (Version 12.0). The 3D geometric mapping of the polarity TPSA and lipophilicity miLogP characteristics of HCA, guttiferone‐E, guttiferone‐J (surface‐coloring characteristics), and spiramycin was performed using Molinspiration (https://www.molinspiration.com). The SwissADME graphical bioavailability radar charts of HCA, guttiferone‐E, guttiferone‐J, and spiramycin were demonstrated (https://www.swissadme.ch/). POLAR, polarity; SIZE, molecular weight; LIPO, lipophilicity; INSOLU, insolubility; FLEX, flexibility; INSATU, unsaturation. The designated red regions in the radar charts adequately represent these chemical compounds’ pharmacokinetic and bioavailability characteristics.

HCA, guttiferone‐E, guttiferone‐J, and SPM pharmacological drug‐likeness properties and QSAR/ADMET characteristics were predicted using Molinspiration, admetSAR2, and SwissADME web servers. The OH‐ and NH‐bond–occupied surfaces of chemical compounds are represented by the topological polar surface area (TPSA), a physicochemical property. As a good predictor of gastrointestinal (GI) absorption, the typical appropriate levels of TPSA positively correlate with the chemical compounds’ H‐bonding potentials. According to previous research, ligands/leads with TPSA values ≥ 140 Å^2^ exhibit weak human GI absorption, and those with TPSA values < 90 Å^2^ are required for blood–brain barrier (BBB) passage and neuronal receptor binding [33–35]. The TPSA values of HCA, guttiferone‐E, and guttiferone‐J were **152.36**, **111.90**, and **91.67 Å** ^**2**^ **,** respectively, compared to SPM (**195.38 Å** ^**2**^), which reflected the diversity of polarity, water solubility, lipophilicity, drug oral bioavailability, BBB permeation, and human GI absorption of HCA, guttiferone‐J, and SPM. A novel study noted that the drug’s human GI absorption is strongly associated with a rotatable bond count of ≤ 10 and a TPSA of ≤ 140 Å^2^ [35, 36], which correlated with the HCA rotatable bond count. The TPSA values of (**5** and **152.36 Å** ^**2**^), guttiferone‐E (**10** and **111.90 Å** ^**2**^), and guttiferone‐J (**10** and **91.67 Å** ^**2**^) compared to SPM (**11** and **195.38 Å** ^**2**^), respectively. In terms of lipophilicity indices, values of log Po/w < +5 indicate a rise in the affinity of drugs for the aqueous phase (raising lipophilicity/hydrophilicity). In contrast, values of log Po/w > +5 indicate restrictions on drug penetration and absorption across biomembranes (raising lipophilicity/hydrophobicity) [33, 36]. This is evident in the lipophilicity indices of HCA (highly lipophobic/hydrophilic), guttiferone‐E (strongly lipophilic/hydrophobic), and guttiferone‐J (strongly lipophilic/hydrophobic) in comparison with SPM (moderate lipophilic/hydrophobic). Figure 1 shows the 3D geometric mapping (surface‐coloring characteristics) of the polarity (TPSA) and lipophilicity (miLog*P*) of HCA, guttiferone‐E, and guttiferone‐J compared to SPM (https://www.molinspiration.com). The features of the graphical SwissADME drug bioavailable radar charts for HCA, guttiferone‐E, guttiferone‐J, and SPM included lipophilicity, insolubility, favorable and safe size, polarity, instauration, and flexibility (Figure 1) (https://www.swissadme.ch/).

Log *Kp* values > 2.5 indicate reduced skin permeation, a characteristic of the putative, selective restorative chemicals [37]. The permeation of skin log *Kp* (cm/s) values of HCA, guttiferone‐E, and guttiferone‐J were **−9.39**, **−2.67**, and **−2.26**, respectively, compared to the **−10.34** log *Kp* value of SPM. Furthermore, HCA, guttiferone‐E, and guttiferone‐J exhibited distinct physicochemical properties and pharmacokinetic characteristics compared with SPM (Table 1). Lipinski’s RO5, the molecular weight (MW) < 500 g/mol, the estimated octanol/water partition coefficient (logP) < +5, the number of H‐bond donors (HBDs) < 5, the H‐bond acceptors (HBA) < 10, and the TPSA value < 140 Å^2^ all indicate that the promising pharmaceutical compounds, when taken orally, will render active and well‐bioavailable [33, 34, 38]. These factors determined and predicted the physicochemical characteristics of HCA, guttiferone‐E, and guttiferone‐J compared with SPM. The biologically active scores of HCA, guttiferone‐E, and guttiferone‐J were evaluated in comparison with the features of SPM using their physicochemical features, drug‐likeness scores, and lipophilicity indices, including MW, HBA, HBD, TPSA, log P, and violations of Lipinski’s RO5 (Table 1).

**TABLE 1:** Predictive QSARs/ADMET in silico properties of *Garcinia cambogia* active phytochemicals compared with the characteristics of spiramycin.

Physicochemical properties	HCA	Guttiferone‐E	Guttiferone‐J	Spiramycin
150 < MW < 500 (g/mol)	208.12	602.81	586.81	843.05
#Heavy atoms	14	44	43	59
#Aromatic heavy atoms	0	6	6	0
0.25 < Fraction Csp3 (insaturation) < 1	0.50	0.50	0.50	0.86
0 < #Rotatable bonds (flexibility) < 9	5	10	10	11
#H‐bond acceptors < 10	8	6	5	16
#H‐bond donors < 5	5	3	2	4
Molar refractivity (MR)	38.63	180.06	178.03	218.97
20 < Polarity TPSA < 130 Å^2^	152.36	111.90	91.67	195.38
Lipophilicity (log *P*_*o*/*w*_)				
iLOGP	−1.05	4.76	5.16	5.61
−0.7 < XLOGP3 < +5	−2.56	10.29	10.73	1.55
WLOGP	−2.28	8.76	9.20	2.33
MLOGP	−2.27	3.78	4.32	−0.95
SILICOS‐IT LOGP	−2.27	9.15	9.61	−0.44
Consensus LOGP	−2.08	7.35	7.80	1.62
Water solubility				
−6 < LogS (ESOL) < 0	0.81	−9.50	−9.68	−5.32
LogS (Ali)	−0.09	−12.58	−12.61	−5.26
LogS (SILICOS‐IT)	2.64	−8.16	−8.76	−1.25
Solubility class (LogS scale)	High soluble	Poor soluble	Poor soluble	Moderate soluble
Pharmacokinetics				
Human oral bioavailability	High	Low	Low	Very low
Human GI absorption	Low	High	High	Low
Caco‐2 permeability	Low	Low	Low	Low
Skin permeation log *Kp* (cm/s)	−9.39	−2.67	−2.26	−10.34
P‐gp inhibitor	No	Yes	Yes	Yes
P‐gp substrate	No	Yes	Yes	Yes
BBB permeation	Low	Moderate	High	No
Subcellular localization	Mitochondria	Mitochondria	Mitochondria	Mitochondria
Glucocorticoid receptor binding	No	Yes	Yes	Yes
Androgen receptor binding	No	Yes	Yes	Yes
Aromatase binding	No	Yes	Yes	No
Estrogen receptor binding	No	Yes	Yes	No
Thyroid receptor binding	No	Yes	Yes	Yes
PPARγ signaling	No	Yes	Yes	Yes
CYP1A2 inhibitor	No	Yes	No	No
CYP2C19 inhibitor	No	Yes	No	No
CYP2C9 inhibitor	No	Yes	No	No
CYP2C9 substrate	Yes	No	No	No
CYP2D6 inhibitor	No	No	No	No
CYP2D6 substrate	No	No	No	No
CYP3A4 inhibitor	No	No	No	No
CYP3A4 substrate	No	Yes	Yes	Yes
OATP1B1 inhibitor	Yes	Yes	Yes	Yes
OATP1B3 inhibitor	Yes	Yes	Yes	Yes
Renal OCT2 inhibitor	No	No	No	No
Drug‐likeness scores				
Lipinski filter #violations	Yes; 0	Yes; 1	No; 2	No; 2
Ghose filter #violations	No; 2	No; 4	No; 4	No; 3
Veber filter #violations	No; 1	Yes; 0	Yes; 0	No; 2
Egan filter #violations	No; 1	No; 1	No; 1	No; 1
Muegge filter #violations	No; 2	No; 2	No; 1	No; 3
Bioavailability score	0.11	0.56	0.56	0.17
Medicinal chemistry friendliness				
PAINS #alerts	0	1	0	0
Brenk #alerts	0	6	5	1
Lead‐likeness #violations	1	3	3	No; 2
Synthetic accessibility	2.93	6.88	6.75	9.87
Toxicological features				
Acute oral toxicity	Class III	Class III	Class III	Class IV
Ames mutagenesis	No	No	Yes	No
Carcinogenicity (binary/trinary)	No	No	No	No
Eye corrosion	No	No	No	No
Eye irritation	Yes	No	No	No
Skin irritation	Yes	No	No	No
Fish aquatic toxicity	No	Yes	Yes	Yes
Honey bee toxicity	No	No	No	No
Hepatotoxicity	Yes	Yes	Yes	Yes
Mitochondrial toxicity	No	Yes	Yes	Yes
Micronuclear	Yes	No	No	Yes
Nephrotoxicity	No	No	Yes	No
Reproductive toxicity	No	Yes	Yes	Yes
Respiratory toxicity	No	Yes	Yes	Yes
skin sensitization	No	Yes	No	No
Biodegradation	Yes	No	No	No

*Note:* #, number; Log*P*, logarithm of partition coefficient between *n*‐octanol and water; FCsp3, total number of sp3 carbons ÷ total carbon count; pharmacokinetics mean absorption, distribution, excretion, metabolism, and toxicity (ADMET) potentials; P‐gp, P‐glycoprotein.

Abbreviations: BBB, blood–brain barrier; GI, gastrointestinal; MW, molecular weight; TPSA, topological polar surface area.

Several studies reported that GC crude extract phytochemicals and/or HCA supplements had no significant toxic effects. In humans, administration of 1667.3 mg/day GC extract (1000 mg HCA/day) for 12 weeks did not indicate a significant adverse impact on serum sex hormones. The oral LD50 values of HCA and HCA lactone were > 5000 mg/kg/day with no drug‐related mortality and toxicity features [26, 39]. According to several biochemical and histopathological analyses, the administration of HCA, HCA lactone, and/or GC crude extract (65% HCA) was safe. It did not cause inflammatory responses, hepatotoxicity, chromosome aberrations, or genotoxicity. However, the consumption of formulations containing GC fruit extract active ingredients with multiple herbal natural ingredients, the use of GC in addition to other medications, or the long administration of high doses of GC phytochemicals induced toxicological features such as toxicity toward spermatogenesis, testicular atrophy, hepatotoxicity, acute liver injury, and ocular complications [39–44]. Hence, these findings confirmed the predictive QSAR/ADMET models and toxicological features of the selective and promising antitoxoplasmosis‐correlated GC active components, such as HCA, guttiferone‐E, and guttiferone‐J (Table 1), compared to SPM.

**FIGURE 2:**
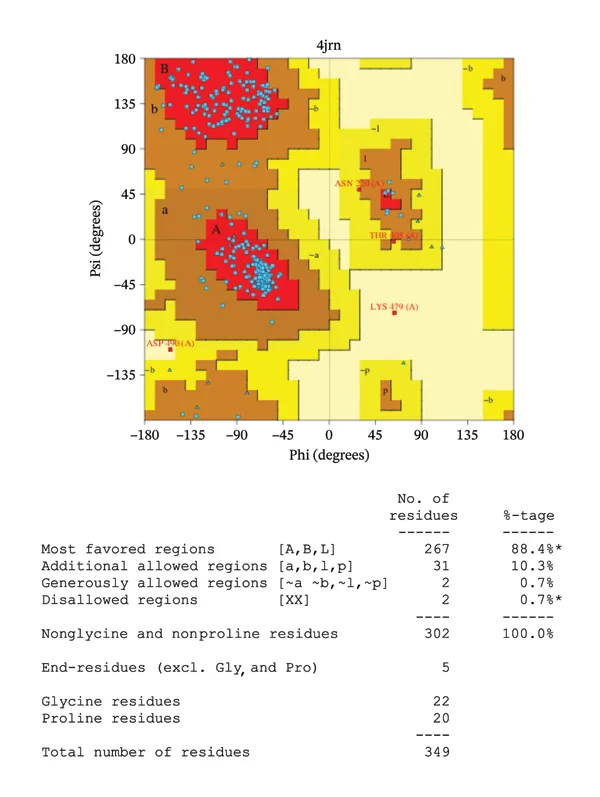
The Ramachandran plot and its statistics demonstrate a high‐quality stability model for the promising antitoxoplasmosis‐related ROP18 transferase target (https://www.ebi.ac.uk/thornton-srv/software/PROCHECK/). Red represents the most preferred sites (A, B, L), brown color represents additional permitted sites (a, b, l, p), bright yellow color represents generously permitted sites (∼a, ∼b, ∼l, ∼p), and light‐yellow color represents outlawed sites (XX). The selective antitoxoplasmosis‐related target is shown as follows: rhoptry protein kinase domain/ROP18 transferase (4JRN).

The selective antitoxoplasmosis ROP18 transferase targets Ramachandran plots, as shown in Figure 2. It is suggested that 88.4% of the ROP18 transferase amino acid residues are located in the most stable favored regions (Figure 2).

**FIGURE 3:**
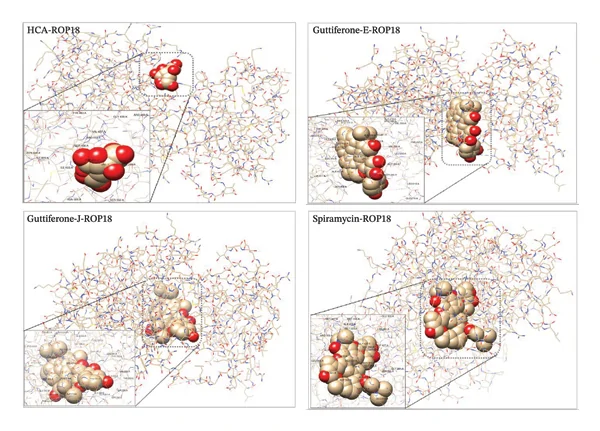
The 3D docked complexes of the highest binding modes of *G. cambogia* active phytochemicals, such as HCA, guttiferone‐E, and guttiferone‐J, toward the potent, selective antitoxoplasmosis‐correlated ROP18 transferase target, compared with spiramycin, a conventional synthetic standard antitoxoplasmosis drug.

For the selected GC phytochemical–correlated antitoxoplasmosis ROP18 complexes, the best favored binding mode/pose was estimated based on the least estimated free energy of binding (Figure 3).

### 3.2. Detection of the Affinity Binding, Inhibition Constant, and the Noncovalent Intermolecular Interactions of the Selective GC Phytochemicals Correlated With *T. gondii* ROP18 Complexes

**FIGURE 4:**
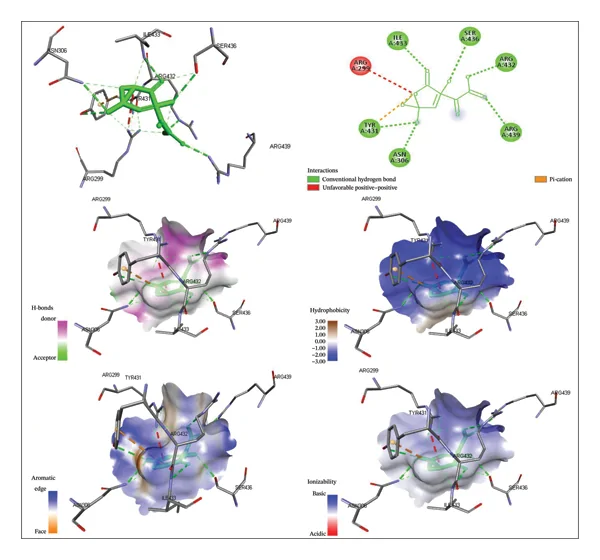
The 3D and 2D docked structures (the highest binding mode), along with the modeling features of the SB‐pharmacophore (H‐bond donor/acceptor, aromaticity, ionizability, and hydrophobicity) for the promising HCA‐correlated ROP18 complex, show the active amino acid residues of the ROP18 transferase, as visualized in BIOVIA Drug Discovery Studio. The intermolecular interactions of H‐bonding (conventional, π‐donor, and carbon), electrostatic (π‐sulfur, π‐anion, and π‐cation), and hydrophobic (π–π stacked, π–π T‐shaped, alkyl, π‐alkyl, π–σ, and amide‐π stacked) were illustrated.

**FIGURE 5:**
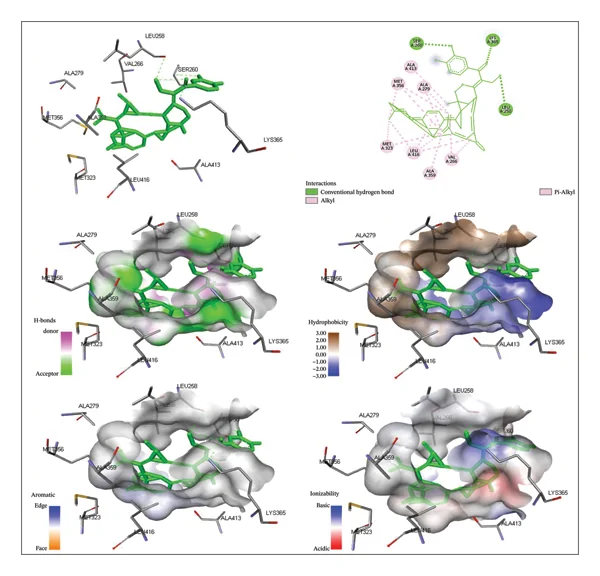
The 3D and 2D docked structures (the highest binding mode), along with the modeling features of the SB‐pharmacophore (H‐bond donor/acceptor, ionizability, hydrophobicity, and aromaticity) for the promising guttiferone‐E–correlated ROP18 complex, are visualized using Visualizer software in BIOVIA Drug Discovery Studio. The intermolecular interactions of H‐bonding (conventional, π‐donor, and carbon), electrostatic (π‐sulfur, π‐anion, and π‐cation), and hydrophobic (π–π stacked, π–π T‐shaped, alkyl, π‐alkyl, π–σ, and amide‐π stacked) were illustrated.

**FIGURE 6:**
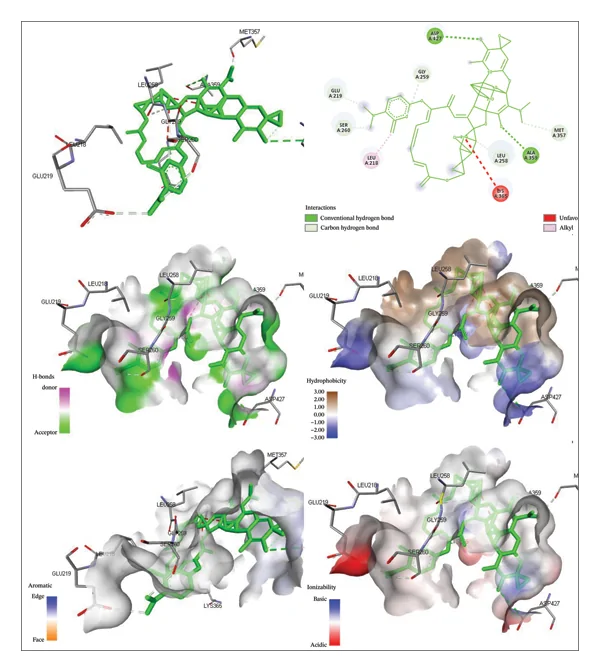
The 3D and 2D docked structures (the highest binding mode), along with the SB‐pharmacophore modeling features (H‐bond donor/acceptor, ionizability, hydrophobicity, and aromaticity) of the promising spiramycin‐correlated ROP18 complex, identify the active amino acid residues of the ROP18 transferase using BIOVIA Drug Discovery Studio Visualizer. The intermolecular interactions of H‐bonding (conventional, π‐donor, and carbon), electrostatic (π‐sulfur, π‐anion, and π‐cation), and hydrophobic (π–π stacked, π–π T‐shaped, alkyl, π‐alkyl, π–σ, and amide‐π stacked) were illustrated.

Table 2 demonstrates that the estimated binding free energy of HCA, guttiferone‐E, and guttiferone‐J toward *T. gondii* ROP18 transferase target was −7.35, −11.81, and −8.03 Kcal/mol, respectively. The estimated binding free energy of SPM to *T. gondii* ROP18 transferase target was −12.99 Kcal/mol. Additionally, the calculated values of inhibition constant (Ki) of HCA, guttiferone‐E, and guttiferone‐J toward the selective *T. gondii* ROP18 transferase target were 4.11 μM, 2.19 nM, and 1.30 μM, respectively. The estimated Ki value of SPM toward the selective *T. gondii* ROP18 transferase target was 301.38 pM. The various noncovalent interactions of HCA, guttiferone‐E, and SPM with the active amino acid residues of their selective and potential antitoxoplasmosis ROP18 target are clearly shown in Figures 4, 5, 6. These interactions included electrostatic (π‐cation; π‐anion; π‐sulfur), hydrophobic (π–π stacked; π–π T‐shaped; alkyl; π–alkyl; π–σ; amide–π stacked), and H‐bonding (conventional; carbon; π‐donor). The intermolecular interactions between the chemical compounds and their particular and potential targets, such as electrostatic and hydrophobic interactions and classical and/or nonclassical H‐bonds, are strongly correlated with the binding modes’ strength, as indicated by the scoring functions [45], which amply supported this in silico research. As shown in Figures 4, 5, 6 and reported in Table 3, the promising GC phytochemical–correlated antitoxoplasmosis ROP18 complexes clearly revealed the various types, strengths, and bond lengths of the intermolecular interactions of HCA and guttiferone‐E with the key amino acid residues of the ROP18 transferase, compared to the selected SPM‐correlated antitoxoplasmosis ROP18 complex. Additionally, in contrast to the SPM‐docked complex, the HCA‐ and guttiferone‐E–interacted complexes may have shown a variety of conventional and carbon H‐bonding, hydrophobic, and electrostatic contacts with the potential antitoxoplasmosis ROP18 transferase (Figures 4, 5, 6 and Table 3). Additionally, the SB‐pharmacophore modeling properties (HBD, HBA, aromaticity, hydrophobicity, and ionizability) of the selective and promising guttiferone‐E–, SPM‐, and HCA‐correlated antitoxoplasmosis ROP18 complexes are comprehensively displayed in Figures 4, 5, 6. Table 3 provides a clear report on the characteristics of intermolecular interactions of H‐bonding, including H‐bond interaction order, HBD, HBA, and H‐bond distance (Å). The creation of drugs that specifically target biological activities, chemical and biological processes, and molecular recognition is significantly aided by H‐bonding, which increases ligand‐binding affinity toward their intended targets [35].

**TABLE 2:** The scoring binding (kcal/mol), binding affinity (Ki), and the stability (RMSD‐tolerance) of the selective *Garcinia Cambogia* phytochemical–correlated antitoxoplasmosis ROP18 transferase complexes were evaluated compared to spiramycin.

Antiparasitic‐correlated targets	Binding scoring (kcal/mol)	Binding affinity (Ki)	RMSD‐tolerance (rmstol) of 2.00 Å
HCA	−7.35	4.11 μM	0.62
Guttiferone‐E	−11.81	2.19 nM	0.86
Guttiferone‐J	−8.03	1.30 μM	1.00
Spiramycin	−12.99	301.38 pM	0.76

*Note:* The values of Cα‐RMSD‐tol and root‐mean‐square deviation reflect the stability of protein–ligand complex conformations.

**TABLE 3:** Characteristics of the H‐bond interactions in the promising and selective *Garcinia cambogia* phytochemical–correlated antitoxoplasmosis ROP18 target complexes compared to spiramycin.

Selective antitoxoplasmosis leads	H‐bond interaction order	H‐bond donor	H‐bond acceptor	H‐bond distance (Å)
HCA	**6** Conventional H‐bond	A:ASN306:ND2	Ligand (O)	2.80
		A:TYR431:OH	Ligand (O)	3.26
		A:ARG432:NE	Ligand (O)	2.77
		A:ILE433:N	Ligand (O)	2.71
		A:SER436:OG	Ligand (O)	2.85
		A:ARG439:NH1	Ligand (O)	2.77

Guttiferone‐E	**4** Conventional H‐bond	A:SER260:N	Ligand (O)	2.96
		A:LYS365:NZ	Ligand (O)	2.76
		Ligand (O)	A:LEU258:O	2.76
		Ligand (O)	A:SER260:O	3.20

Spiramycin	**3** Conventional H‐bond **5** Carbon H‐bond	A:ALA359:N	Ligand (O)	2.61
		Ligand (O)	A:ASP427:OD1	3.40
		Ligand (O)	A:ALA359:O	2.82
		A:GLY259:CA	Ligand (O)	3.33
		Ligand (C)	A:LEU258:O	3.80
		Ligand (C)	A:SER260:O	2.85
		Ligand (C)	A:GLU219:OE1	2.76
		Ligand (C)	A:MET357:O	2.75

*Note:* The active essential amino acid residues (active catalytic‐binding pocket) were reported.

### 3.3. Parasitological Results

**FIGURE 7:**
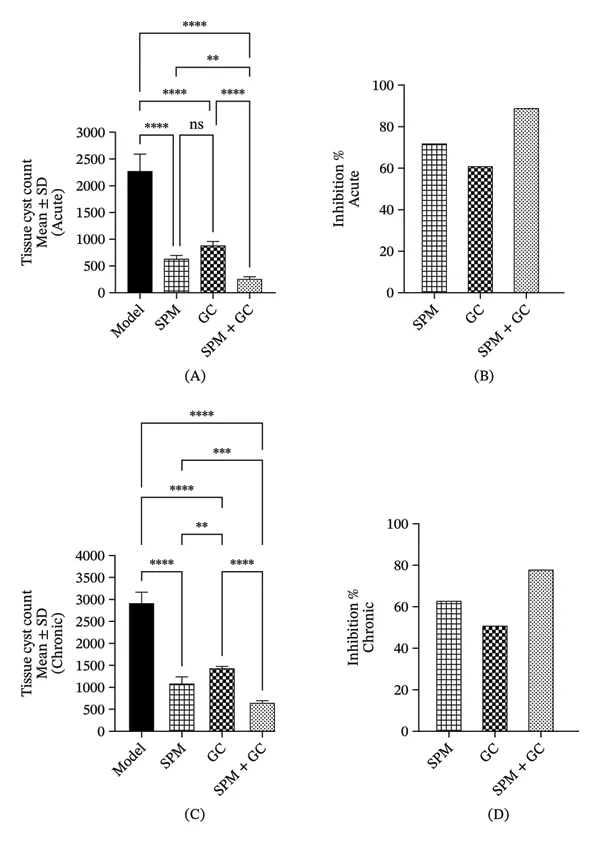
(A) represents the number of *T. gondii* brain cysts, and (B) represents the inhibition percentage of *T. gondii* brain cysts in the experimental groups of acute toxoplasmosis. (C) represents the number of *T. gondii* brain cysts, and (D) represents the inhibition percentage of *T. gondii* brain cysts in the experimental groups of chronic toxoplasmosis. Data are expressed as mean ± SD, and differences were regarded as significant at *p* < 0.05^∗^, *p* < 0.01^∗∗^, *p* < 0.001^∗∗∗^, and *p* < 0.0001^∗∗∗∗^ or nonsignificant (ns). The inhibition % was calculated as follows: = [(the model group (Mean count) − the treated group (mean count))/the model group (mean count)] × 100.

*Toxoplasma* cyst counts and the percentage of inhibition in different experimental groups are displayed in Figure 7. In terms of treatment during acute toxoplasmosis, the number of *Toxoplasma* cysts in the treated groups (SPM (636.2 ± 58.24), GC (880.8 ± 76.58), and SPM + GC (258.3 ± 42.5)) was significantly reduced compared with the model group (2262 ± 330.2) (*p* < 0.001). The treatment inhibition percentages in the SPM‐, GC‐, and SPM + GC–treated groups were 72%, 61%, and 89%, respectively, with the SPM + GC–treated group demonstrating the best outcomes.

Regarding treatment during chronic toxoplasmosis, the number of *Toxoplasma* cysts in the treated groups (SPM (1087 ± 157.1), GC (1435 ± 42.31), and SPM + GC (650 ± 45.06)) was significantly reduced compared with the model group (2911 ± 253.8) (*p* < 0.001). The treatment inhibition percentages in SPM‐, GC‐, and SPM + GC–treated groups were 63%, 51%, and 78%, respectively, with the SPM + GC–treated group demonstrating the best outcomes.

### 3.4. Histopathological Results

**FIGURE 8:**
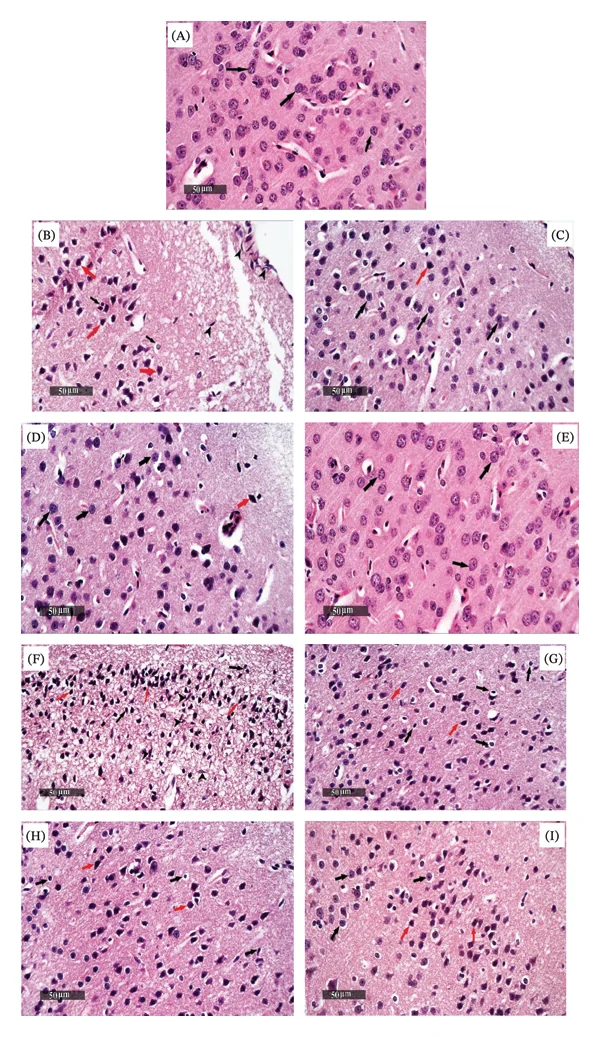
Photomicrograph of brain tissue. (A) Group I of healthy mice with intact neurons (black arrows). (B) Group II of acutely infected model mice showed numerous shrunken, pyknotic neurons (red arrows), free tachyzoites in the submeningeal space and the interneuronal brain matrix (arrowheads), and reactive glial cell infiltrates (black arrows). (C) Group III of acutely infected SPM‐treated mice had a few focal areas of degenerating neurons (red arrow) and more abundant records of intact neurons (black arrows). (D) Group IV of acutely infected GC‐treated mice showed a few focal areas of degenerating neurons (red arrow), with apparently intact neurons (black arrows) observed. (E) Group V of acutely infected GC + SPM–treated mice showed apparently intact, well‐organized neurons across different layers (black arrows). (F) Group II of chronically infected model mice demonstrated numerous records of shrunken, pyknotic neurons in the outer cortical layers (red arrows). Higher numbers of free tachyzoites were observed compared with acute infection (arrowheads), accompanied by greater reactive glial cell infiltrates (black arrows). (G) Group III of chronically infected SPM‐treated mice showed a moderate number of apparently intact neurons and moderate glial cell infiltrates (black arrows), interspersed with a few instances of neuronal damage and degenerative alterations (red arrows). (H) Group IV of chronically infected GC‐treated mice showed persistent neuronal damage and degenerative changes (red arrows), alternating with a few apparent intact neurons and moderate glial cell infiltrates (black arrows). (I) Group V of chronically infected GC + SPM–treated mice showed abundant records of intact neurons (black arrows), and a few focal records of degenerated neurons were shown (red arrows).

Histopathological analysis of brain tissue sections from mice in division A receiving treatment during acute toxoplasmosis is displayed in Figure 8. Group 1, which contained healthy mice, demonstrated typical histological features of cortical layers, with apparently intact, well‐organized neurons across layers (Figure 8A). On the other hand, Group II, which contains model mice, showed severe, diffuse evidence of neuronal degenerative alterations, with numerous records of shrunken, pyknotic neurons in the outer cortical layers. Focal records of free tachyzoites were demonstrated in the submeningeal space and interneuronal brain matrix, accompanied by moderately higher reactive glial cell infiltrates (dashed arrow) and marked vacuolization of the brain matrix (Figure 8B). In contrast, the SPM‐, GC‐, and SPM + GC–treated groups showed improvement and a return to typical histological structures compared with the model mice. The SPM (Figure 8C) and GC (Figure 8D) treatment groups showed moderate neuronal protection, with few focal areas of degenerating neurons. More abundant records of apparently intact neurons were observed, and intact interneuronal brain matrix was shown with minimal edema, vacuolization, and sporadic glial cell infiltrates. The SPM + GC–treated group showed the greatest improvement, with apparently intact, well‐organized neurons across layers. Furthermore, an intact intercellular matrix was observed, with minimal reactive glial cell infiltrates (Figure 8E). Similarly, the histopathological analysis of brain tissue sections from mice in division B, which received treatment during chronic toxoplasmosis, is displayed in Figure 8F, G, H, and I.

### 3.5. Immunohistochemical Results

**FIGURE 9:**
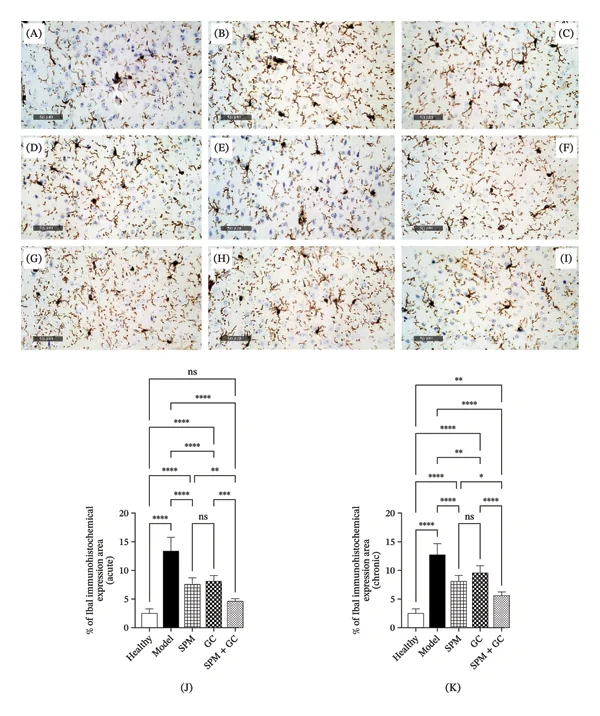
Iba1 immunohistochemical expression in the brain tissue of different experimental groups. (A) Group I (healthy mice) in both divisions showed low Iba1 expression. Division A: (B) GII (model mice) showed an elevation in the Iba1 level; C–E: treated groups showed that Iba1 expression is downregulated. Division B: (F) GII (model mice) showed an elevation in the Iba1 level; G–I: treated groups showed that Iba1 expression is downregulated. Semiquantitative estimation of Iba1 expression score for (J) Division A and (K) Division B. The results are shown as mean ± SD, ^∗^ *p* < 0.05, ^∗∗^ *p* < 0.01, ^∗∗∗^ *p* < 0.001, ^∗∗∗∗^ *p* < 0.0001.

In both divisions A and B, Iba1 expression was low in Group 1 (healthy mice) (Figure 9A, J, K). Still, it was significantly higher in the brains of GII (model mice) for divisions A (Figure 9B) and B (Figure 9F). In both divisions, compared with the model GII, a substantial decline in Iba1 expression was observed in all treated groups (GIII, GIV, and GV; *P* < 0.0001). In GV treated with both SPM and GC, the lowest Iba1 level was observed (Figure 9).

### 3.6. Integrative Analysis of Parasitological and Immunohistochemical Data

**FIGURE 10:**
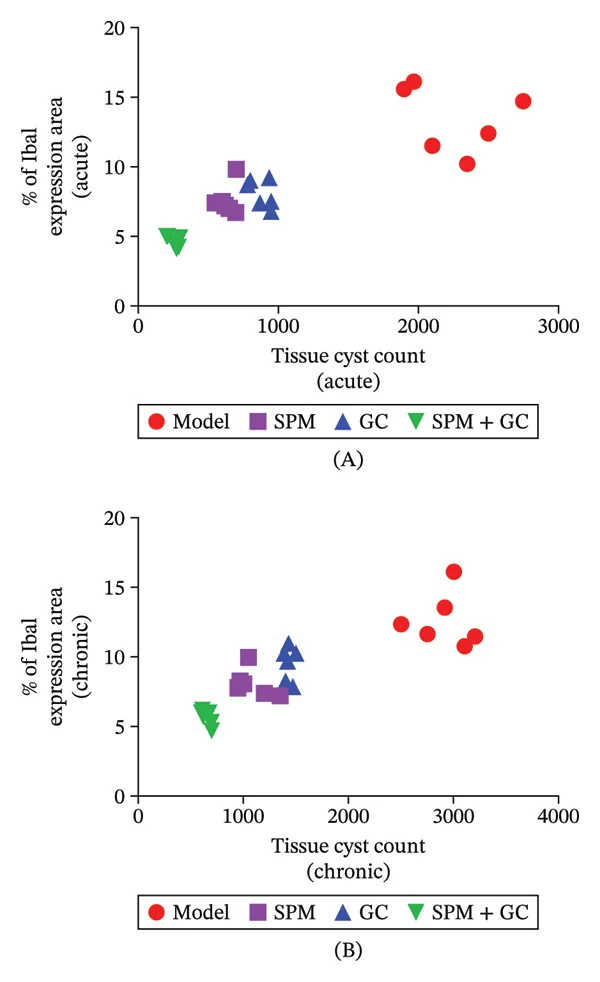
Scatter plot of tissue cyst count versus % Iba1 expression area with Spearman correlation between tissue cyst burden and Iba1 expression. (A) Acute infection: (*r* = 0.8546, *p* < 0.0001), (B) chronic infection: (*r* = 0.8762, *p* < 0.0001).

To quantitatively integrate parasitological and immunohistochemical data, we examined the relationship between tissue cyst burden and microglial activation, as reflected by Iba‐1 expression. In the acute infection groups, Spearman’s rank correlation revealed a strong positive association between tissue cyst count and % Iba‐1 expression area (*r* = 0.8546, 95% CI = 0.6819–0.9371, *p* < 0.0001, *n* = 24, Figure 10A).

A similarly strong correlation was observed in the chronic infection groups (*r* = 0.8762, 95% CI = 0.7254 to 0.9468, *p* < 0.0001, *n* = 24, Figure 10B). Thus, animals with higher tissue cyst burdens displayed markedly increased Iba‐1 expression, whereas those receiving SPM, GC, or their combination exhibited both reduced parasite counts and lower Iba‐1 expression levels.

## 4. Discussion

To identify biological signaling pathways and make precise, reliable predictions in both in vivo and in vitro research, the bioinformatics specialty integrates computational technologies [46]. The putative compounds’ conformations and orientations within the active‐binding pockets of their specific and potential targets are identified using molecular docking simulations. Conformations generated by search algorithms are graded according to their scoring functions [10, 45]. Conventional synthetic antiparasitic drugs induce strong oxidative stress responses, generate free radicals, and disrupt cellular redox homeostasis. SPM is a potent macrolide antibiotic, and its characteristics are positively correlated with its high long‐term toxicological features, low human oral bioavailability, GI absorption, Caco‐2 permeability, and its low BBB and placental barrier penetration (PBP), which potentially reduce its restorative properties against brain and/or fetal and neonatal toxoplasmosis [47]. The findings of Ref. [41] confirmed the LB‐virtual screening characteristics of SPM, as previously reported [2]. As a native Southeast Asian fruit, GC L. (Malabar tamarind) is used as a food additive and preservative, as well as a spice, bulking agent, and flavoring agent, and is considered a popular traditional medicinal plant [44]. GC fruits and their rinds mainly contain organic acids like HCA (52.4% of GC) that potentially have weight reduction, hypolipidemic, hypoglycemic, antiobesogenic, and appetite‐suppressant activities [39]. Furthermore, GC fruits and their rinds include other organic acids as HCA lactone (*Garcinia* lactone), citric‐, malic‐, and tartaric acids, polyisoprenylated benzophenones as garcinol (guttiferone‐E), isogarcinol (cambogin), guttiferone‐I, guttiferone‐J, guttiferone‐K, guttiferone‐N, and guttiferone‐M, xanthones as garbogiol, oxy‐guttiferone‐I, guttiferone‐K, guttiferone‐K2, and guttiferone‐M, amino acids as arginine, glutamine, leucine, isoleucine, proline, asparagine, and γ‐aminobutyric acid, carbohydrates, phenolics, alkaloids, and flavonoids. Moreover, GC fruits and their rind extract phytochemicals have several potent therapeutic features such as antioxidant, anti‐inflammatory, antiulcerogenic, antiseptic, antidiarrhea, anticancer, hepatoprotective, neuroprotective, antimicrobial, antiviral, antiparasitic, antiobesity, antidiabetic, hypolipidemic, hypoglycemic, antiadipogenic, weight‐loss properties, and appetite‐suppression effects [43, 48, 49].

In this research, predictive QSAR/ADMET characteristics and SB‐molecular in silico docking simulations were used to detect, evaluate, and present QSAR models, binding affinities, and estimated inhibition constant values for HCA, guttiferone‐E, guttiferone‐J, and SPM against the selective *T*. *gondii* ROP18 transferase target. The HCA‐, guttiferone‐E–, guttiferone‐J–, and SPM‐correlated antitoxoplasmosis ROP18 complexes’ binding affinities, scoring functions, and intermolecular noncovalent interactions demonstrated the potency of the target–ligand binding activities, the conformations’ binding stability, and the variety of the intermolecular interactions in the current study [50], which supported the study’s outcomes.

Binding‐mode efficacy and inhibitory potential are strongly correlated with the number of H‐bonds [51]. The enzyme half‐maximum inhibitory concentration (Ki) of a particular inhibitor is known as the estimated inhibition constant. It assesses the ability of selective ligands to downregulate enzymes’ biological functions. The present investigation’s outcomes clearly show that when Ki values are < 100 μM, the chemical compounds are considered potent inhibitors. In comparison, when Ki values are > 100 μM, the inhibitors are considered nonpotent. The strongest connections between the ligand’s end OH/NH ions in the amino acids of the target protein are H‐bonds. In the target–ligand‐docked complexes, the number of ligand OH groups is potentially associated with the number of H‐bonding interactions and their binding affinities and scoring functions against the selective and promising proteins of interest [51], which is obviously revealed in the 2D cleaned chemical structures and complex forms of HCA, guttiferone‐E, and guttiferone‐J compared to SPM.

ROP18, a secreted serine/threonine rhoptry kinase, was selected as a target not on the basis of new experimental data in our study, but because extensive prior work has identified it as a key virulence factor and an attractive drug target [52, 53]. In murine models, the expression and catalytic activity of ROP18 are strongly correlated with virulence; ROP18‐deficient mutants exhibit significantly lower lethality and decreased intracellular proliferation [52, 53]. Mechanistically, ROP18 targets host signaling proteins, such as NF‐κB p65, and phosphorylates and deactivates host immunity‐related GTPases at the parasitophorous vacuole membrane, preventing IFN‐γ‐dependent vacuole destruction and reducing proinflammatory responses [54]. The ROP18 kinase domain has been structurally resolved, revealing both the ATP‐binding site and an additional ligand‐binding pocket required for virulence, which has already been exploited in virtual screening and docking studies to identify small‐molecule ROP18 inhibitors and pharmacophore models [55].

*T. gondii* is a major obligate intracellular protozoan parasite that infects, invades, and replicates within all nucleated mammalian cells [56–58]. *T. gondii* ROP18 transferase contains a serine/threonine protein domain. The kinase domain of *T. gondii* ROP18 transferase is considered an essential virulence factor that strongly associates with *T. gondii* infection, invasion, and pathogenesis. Rhoptry proteins, ROP proteins, are rhoptries/kinases secreted from the conoids/Rhoptry organelles of the *T. gondii* parasite. ROP proteins, such as the *T. gondii* ROP18 transferase, are positively correlated with *T. gondii* parasite internalization, manipulation of the host innate and adaptive immune responses, and cell apoptosis [59–61]. The structure of the *T. gondii* ROP18 transferase has conserved several kinase‐active residues. The catalytic‐binding cavities include the active residues Lys281, Asp409, and Asp427. The glycine‐rich loop (G‐loop) binds the ATP substrate at the active site, which comprises the residues Val266, Phe263, Gly259, Gly261, and Gly262. The A‐loop of ROP18 transferase includes Phe428 and Gly429 residues that regulate the enzymatic action of ROP18. Asn414 and Lys411 are located in the catalytic loop, while Ala359 is situated in the hinge region. As a gatekeeper, Met356 covers the hydrophobic‐binding pocket. Furthermore, Glu300 is considered a key amino acid residue that forms a salt bridge with Lys281, facilitating ATP stabilization and the enzymatic activity of the ROP18 transferase [59]. Moreover, *T. gondii* ROP18 transferase widely includes important active catalytic‐binding regions such as the disordered linker (211–223 residues), G‐loop (258–266 residues), β3‐β5 sheet (276–284 and 353–358 residues), hinge region (359–365 residues), gatekeeper residue (Met323), and catalytic loop (411–416 residues). In the active‐binding pocket of *T. gondii* ROP18 transferase, the 30C thiazolidinone derivative moieties greatly form diversity of intermolecular interactions with the ROP18 key residues that stabilize the ATP‐binding cavity to disturb the enzymatic action of ROP18 transferase, including Lys281, Asp427, Lys411, Ser260, Gly259, Gly261, Gly262, Gly429, Ala264, Ala413, Val266, Phe263, Phe283, Glu300, Asp362, Asn414, Leu416, and Thr430 [59]. All these previous findings clearly confirm and align with the SB‐virtual screening analysis of the selective and promising ROP18 complexes correlated with guttiferone‐E and SPM. Investigations of Ref. [14] reported that a sucrose molecule, a biologically interesting molecule, interacts with *T. gondii* ROP18 transferase surface in an additional potential ligand‐binding cavity outside its catalytic active‐binding sites. Furthermore, the mutational analysis confirms an essential function of this additional region in the *T. gondii* virulence and pathogenicity [14], which clearly visualized and matched with our in silico findings about the intermolecular interactions of HCA with the promising antitoxoplasmosis ROP18 target. As a molecular docking simulation, the ROP18‐HCA docked complex demonstrated a novel and additional ligand‐binding pocket of ROP18 outside its original catalytic active‐binding sites, which was considered a critical site for the ROP18‐mediated virulence and pathogenesis through the noncatalytic mechanisms such as the allosteric regulation.

The ME49 strain was used in the current study because it provides a reliable experimental model that includes both an early acute phase of tachyzoite proliferation and a chronic phase, corresponding to the formation of persistent brain tissue cysts. ME49 allows the evaluation of therapeutic interventions at both stages of infection within the same parasite strain, making it especially appropriate for studies examining the progression from acute to chronic disease, even though the Type I RH strain is the traditional model of fulminant acute toxoplasmosis [62, 63].

Our results revealed a decrease in parasitic load in both acute and chronic infections across all treated groups, with the highest reductions in the group receiving the combination of GC and SPM: 89% and 78% for acute and chronic infections, respectively. In accordance with our findings, treatment with fractions of *G. mangostana* Linn [4] and *G. kola* [64] induced decreases in observed densities of tachyzoites. Moreover, the combination of the *G. mangostan*a extract and chlorhexidine yields the best reduction in the number of *Acanthamoeba* trophozoites [6]. In ours, the drug combination reduces parasitic load, possibly due to the synergistic effect of GC, which interferes with ROP18 transferase and SPM, thereby hindering protein synthesis [65].

In our study, histopathological reports indicate that treatment with either SPM or GC showed a modest protective effect on neurons. In comparison, the group that received SPM + GC showed the greatest improvement, with neurons at various levels appearing intact and well‐organized. These results are consistent with the findings of Ref. [64], who reported that *G. kola* preparations showed promise as a treatment for cerebral toxoplasmosis. The neuroprotective effect of *G. kola* was demonstrated through phytochemical screening, which revealed that its secondary metabolite profile exhibits anti‐inflammatory, antiparasitic, neuroprotective, and antioxidant properties.

Iba1 is a cytoskeletal, actin‐crosslinking protein that is highly conserved and expressed selectively in microglia and related macrophage‐lineage cells in the brain. Hence, its detection by immunohistochemistry is a reliable method for quantitatively identifying and analyzing microglia across different brain regions and species under both physiological and pathological conditions [32]. Regarding central nervous system infections, including *T. gondii*, microglia are key innate immune cells that become activated, produce proinflammatory mediators, and can contribute to synaptic dysfunction and neuronal apoptosis when excessively stimulated. Increased Iba1 staining intensity and area are consistently observed in neuroinflammatory states and serve as surrogate markers of microglial activation [66, 67]. Accordingly, anti‐Iba1 immunostaining was used in the current study to quantify microglial activation in *T. gondii* infection and to evaluate how SPM, GC, and their combination modulate microglia‐mediated neuroinflammation and neuronal injury, thereby directly linking the antibody’s biological relevance to the main pathophysiological mechanisms under investigation.

Regarding the status of microglia cells (expression of Iba1) in the current study, all treated groups, GIII, GIV, and GV, showed a significant decrease in Iba1 expression levels compared to the model group GII (*P* < 0.0001). The lowest Iba1 level was observed in GV, which received treatment with both SPM and GC. Our results are consistent with recent research [68], which demonstrated that acute *T. gondii* infection increases Iba1 expression in microglia. This study also reported that *T. gondii* may activate microglia, leading to neuronal death in an in vitro coculture of neurons and microglia. This alteration was reversed, and neuronal damage in *Toxoplasma*‐infected mice was reduced by treatment with both SPM and GC. Thus, it is believed that the combination of the two drugs reduces inflammatory responses, neuronal damage, and elevated microglial activation caused by acute toxoplasmosis.

Integrative parasitological and immunohistochemical analyses were performed to assess the relationship between tissue cyst burden and Iba1 expression using Spearman’s correlation in acute infection (*r* = 0.8546, *p* < 0.0001) and chronic infection (*r* = 0.8762, *p* < 0.0001). These results suggest that the treatment plans not only reduce the parasitological burden in the brain but also reduce microglial activation, which is a major cause of neuroinflammation and neuronal damage in cerebral toxoplasmosis. GC’s antitoxoplasmosis and neuroprotective effects are supported by the high agreement between parasitological and immunohistochemical endpoints, especially when paired with SPM.

## 5. Conclusion

Thus, this study highlights the benefits of combining GG with SPM in treating and managing *Toxoplasma* infection. As a result, this strategy shows promise as an adjunct in treating toxoplasmosis, as it prevents microglial activation, thereby reducing damage induced by *T. gondii* infection in neurons. A new and extra ligand‐binding pocket of ROP18 outside of its original catalytic active‐binding sites was revealed by the ROP18‐HCA docked complex. This pocket was considered crucial for ROP18‐mediated virulence and pathogenesis. Future research with more updated fractionation techniques could uncover the active principles behind the effects of GC on brain toxoplasmosis and systemic illness linked *to T. gondii*.

## 6. Current Study Limitations and Future Prospects

Although the docking results are predictive and helpful, further in vitro enzyme kinetics are required, as they cannot provide definitive evidence of enzymatic inhibition nor replace experimental validation.

H&E staining was primarily used as a standard first‐line stain in experimental neuropathology to assess the general brain architecture and neuropathological changes, rather than as a specific method for neuronal degeneration. Also, our interpretation of neuronal integrity preservation was not based solely on H&E. Instead, it was based on combined evidence from H&E evaluation of neuronal injury patterns and quantitative immunohistochemistry of Iba1, a microglial activation marker and an indicator of neuroinflammatory burden closely linked to neuronal damage in *T. gondii* infection. Nevertheless, more specific markers of neuronal degeneration and apoptosis, such as Fluoro‐Jade B/D, Nissl staining, TUNEL, or cleaved caspase‐3 immunostaining, are needed to strengthen the pathological characterization of neuronal degeneration.

Although the current study demonstrated significant reductions in brain parasite burden and neuroinflammation in the treated groups, particularly in combination with SPM, it did not include the quantification of tissue cysts using immunofluorescence‐based cyst wall markers (e.g., DBA or BAG1), which would provide further confirmation of the impact of GC on cyst morphology and density.

## Author Contributions

Marwa M. I. Ghallab: conceptualization, data curation, visualization, and writing–original draft, and review and editing. Eman S. El‐Wakil: conceptualization, data curation, visualization, investigation, data curation, methodology, and writing–original draft, and review and editing. Rabab S. Zalat: conceptualization, investigation, methodology, and review and editing. Mina A. Almayouf and Hayat S. Al‐Rashidi: investigation, data curation, visualization, and review and editing. Wafa Abdullah I. Al‐Megrin: investigation, data curation, methodology, and review and editing. Salwa M. Morsy: conceptualization, data curation, visualization, writing original draft, and review and editing.

## Funding

This research has no funding.

## Disclosure

All authors have read and agreed to the published version of this manuscript.

## Conflicts of Interest

The authors declare no conflicts of interest.

## Data Availability

The data supporting this study’s conclusions are freely accessible in figshare at https://doi.org/10.6084/m9.figshare.31006144, Ref. [69].
